# Investigation of return photons from sodium laser beacon excited by a 40-watt facility-class pulsed laser for adaptive optical telescope applications

**DOI:** 10.1038/s41598-018-27576-x

**Published:** 2018-06-15

**Authors:** Qi Bian, Yong Bo, Junwei Zuo, Min Li, Ruoxi Dong, Keran Deng, Dingwen Zhang, Liping He, Qingshuang Zong, Dafu Cui, Qinjun Peng, Hongbin Chen, Zuyan Xu

**Affiliations:** 1 Key Lab of Solid State Laser, Technical Institute of Physics and Chemistry, Chinese Academy of Sciences, Beijing, 100190 China; 2 Key Lab of Functional Crystal and Laser Technology, Technical Institute of Physics and Chemistry, Chinese Academy of Sciences, Beijing, 100190 China; 3 University of Chinese Academy of Sciences, Beijing, 100190 China; 4 Institute of Optics and Electronics, Chinese Academy of Sciences, Chengdu, 610209 China; 5 Yunnan Observatories, Chinese Academy of Sciences, Kunming, 650216 China

## Abstract

The brightness of the artificial beacon is one critical performance parameter for adaptive optics. Here, a 40-watt level narrow-linewidth microsecond pulsed yellow laser is produced at 589 nm with a high repetition frequency of 600 Hz and a pulse duration of 120 μs. An experiment to project the pulse beam up to the sky and measure the fluorescence photon returns of the Na atoms has been held on the 1.8-meter telescope in Lijiang observatory. During the sky test, a laser guide star (LGS) spot is firstly observed with Rayleigh scattering elimination by means of a gateable pulse format. And, the central wavelength of the laser could be accurately locked to be 589.1584 nm with a linewidth of ~0.34 GHz to match that of sodium-D_2a_ line. Optical pumping with circularly polarized light has also been used to increase the brightness of sodium LGS. In order to maximize the return flux, sodium D_2b_ repumping option is done by an electro-optic modulator with the optimum D_2a_-D_2b_ frequency offset. As a result, a bright sodium LGS with the return flux of 1610 photons/cm^2^/s is achieved, corresponding to ~47 photons/cm^2^/s/W of emitted laser power, which represents a significant improvement in terms of brightness reported ever.

## Introduction

The ground-based large-aperture telescopes, as a powerful tool for comprehensive understanding of the Universe, advanced rapidly with higher sensitivity and higher spatial resolution^1^ and achieved significant observations^2^. However, atmospheric turbulence distorts light waves received from space objects and severely limits the imaging resolution of large telescopes. Fortunately, these distortions can be practically eliminated in real time via a technology known as adaptive optics (AO), where AO uses the laser guide stars (LGS) as reference sources to probe atmospheric turbulence and provide feedback to deformable mirrors in order to compensate image blur effects induced by this turbulence^3–5^. Sodium LGS are obtained by illuminating the layer of atomic sodium in the mesosphere at 80–100 km altitude using a yellow wavelength at 589 nm and causing it to fluoresce^6–8^, which is considered best choice of beacons for current 8–10 m class astronomical telescopes including the Very Large Telescope and Keck telescope^9^. However, the effectiveness of a sodium LGS-AO system depends on producing sufficiently bright guide star that the photon return from the laser beacons does not limit the resolution of the wavefront sensor, and this motivates the development of high-power sources tuned to sodium strong D_2a_ absorption line.

Sodium beacon lasers have been widely studied with nonlinear frequency-conversion techniques of fiber lasers^10–12^ and solid-state lasers^13–19^. Moreover, the diode-pumped solid-state lasers have shown well promising with the advantages of higher power, more reliability, and a more robust setup. To date, by using the sum frequency generation (SFG) of diode-pumped Nd:YAG laser, continuous-wave (CW) sodium beacon lasers of more than 50 W have already been successfully developed and deployed for the astronomical community. Compared with the CW format, the quasi-continuous-wave (QCW) pulsed guide star lasers would be of greater significance to gate out unwanted Rayleigh scattering noise from low atmosphere and avoid the fratricide effect in multiple LGS systems^20–22^. However, there have been only a few papers reporting the use of the pulsed yellow laser for sodium LGS generation for astronomical AO application. For example, a macropulse/micropulse, mode-locked sum-frequency laser with a bandwidth of 1 GHz and an output power of 7 W is reported, delivering multiple 2 ns micropulses in a 160 μs long macropulse^16^, and the photon return of 80–560 photons/cm^2^/s, corresponding to 17–120 photons/cm^2^/s/W of emitted laser power, is generated on the 200 inch Hale telescope at Palomar Observatory^23^. Lockheed Martin Coherent Technologies has produced a 50-W level commercial mode-locked sodium laser with a spectral bandwidth of 2.1 GHz, a 77 MHz pulse train and the nominal pulse widths on the order of ~300 ps^17^, and the resulting sodium photon return is in the range of 166 to 257 photons/cm^2^/s (i.e. 23–36 photons/cm^2^/s/W) at the Gemini South 8-m telescope atop Cerro Pachón in Chile^24^. In ns pulse regime, a Q-switched sodium laser offers an output power more than 8 W, a repetition frequency of 5 kHz, a pulse width of 152 ns and the linewidth less than 3.5 GHz, and generating the photon return of ~46 photons/cm^2^/s (i.e. ~6 photons/cm^2^/s/W) in the Laboratory^25^. However, high peak power of those short pulse lasers would easily result in the saturation effect of sodium atoms in the mesosphere, thereby leading the return flux dropping down. The theoretical and experimental investigations suggest that QCW microsecond (μs) long pulse format is extremely well-suited for future sodium LGS-AO system^22^. With a pulse duration of 140 μs, a 20 W class sodium laser is developed with a 50 Hz repetition rate and a linewidth of 0.6 GHz^18^, and the photon return of 80–745 photons/cm^2^/s is obtained (i.e. ~40 photons/cm^2^/s/W)^26^. However, its repetition rate is too low for AO system to detect and correct high-order aberration caused by atmospheric turbulence. Recently, we demonstrate a 500 Hz QCW microsecond-pulse sodium laser with an output power of 33 W, a pulse width of 120 μs and linewidth of 0.44 GHz^19^, and a photon return of 600–900 photons∕cm^2^∕s (i.e. 30–45 photons∕cm^2^∕s/W) is realized on the Large Zenith Telescope facility of the University of British Columbia. However, the pronounced laser spiking in pulsed laser systems cannot be avoided, which induces low return flux efficiency due to high peak power. Most of the above-mentioned 589 nm lasers have been used to create a sodium beacon for AO system, but the brightness is relatively low.

In this letter, we report an all-solid-state, 41 W, sub-GHz line width, hundred-microsecond pulsed 589 nm sodium laser with 600 Hz repetition rate, based on SFG of two QCW diode-pumped Nd:YAG master-oscillator power-amplifier (MOPA) lasers operating at 1319 and 1064 nm, respectively. Compared with our previous work^19^, the spiking behavior of 589 nm laser is efficiently suppressed with the insertion of doublers in each oscillator, which has the effect of avoiding the sodium atoms saturation and improving the coupling efficiency. A prototype of this laser system is mounted on the 1.8 m telescope for astronomical AO application, where improving sodium excitation efficiency by optimizing the pulse format, yellow wavelength, polarization and repumping characteristics of 589 nm laser beam are a focus of study. To keep the laser working at a stable state, the central wavelength of the prototype output is locked to the sodium D_2a_ absorption line at 589.1584 nm, and the observed LGS angular diameter is 4 arcsec without Rayleigh scattering noise. To further increase the return flux from a LGS, a quarter-wave plate is inserted in the laser delivery path to generate a circular polarized beam from a linearly polarized beam for better optical pumping. Moreover, repumping the sodium atoms at D_2b_ line is provided by including a ~10% fraction of the launched power at ~1.71 GHz toward the blue with respect to D_2a_ line of sodium, where a significant improvement in the photon return is demonstrated by this way. As a result, the return flux is measured as high as 1610 photons/cm^2^/s (i.e. ~47 photons/cm^2^/s/W) when a circular polarized light is launched to zenith during the on-sky test, which represents a critical step for the sodium return flux achieved by a pulsed sodium laser over previous work.

## Experimental Details

**Figure 1:**
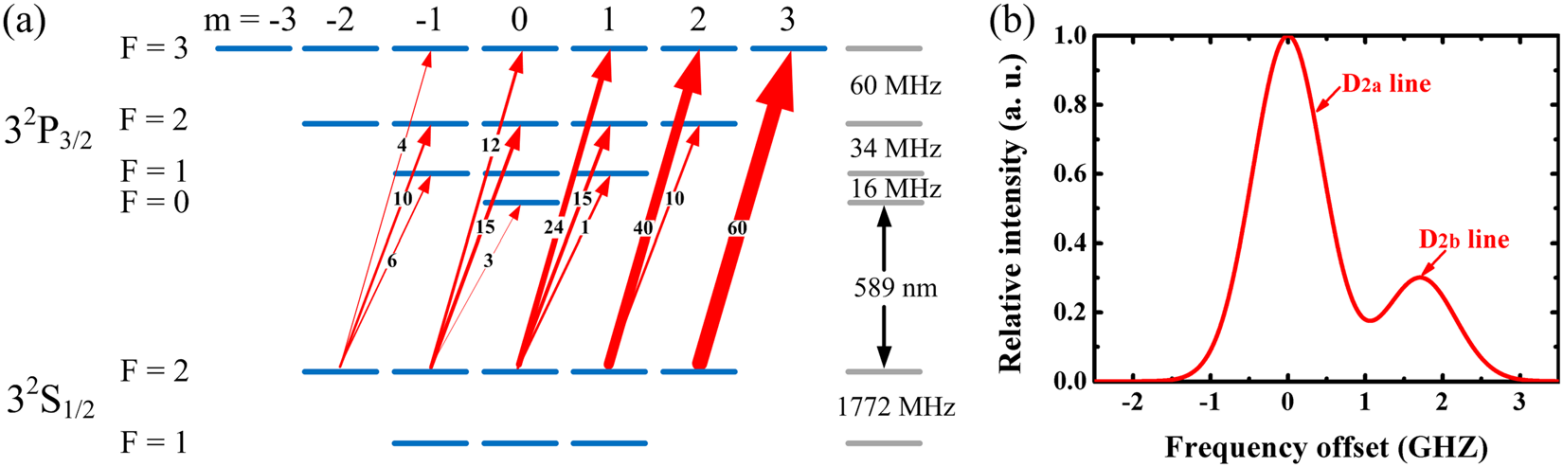
(**a**) Energy level diagram of the sodium D_2_ line, showing D_2a_ transitions that can be produced by irradiation with circularly polarized light inducing ∆M = 1 absorptive transitions. The numbers between the ground and excited states or the widths of the arrows indicate the relative transition strengths. (**b**) Doppler-broadened hyperfine structure of the sodium D_2_ transition at mesospheric temperatures near 190 K. The splitting is almost entirely due to the splitting in the 3^2^S_1/2_ ground state.

Sodium LGS takes advantage of the 3^2^S_1/2_–3^2^P_3/2_ electric dipole transition, known as the D_2_ line. Figure 1(a) illustrates the energy levels of 3^2^P_3/2_ and 3^2^S_1/2_ involved in the sodium D_2_ transitions^27^. The ^2^S ground state consists of two hyperfine multiplets, F = 1 and 2, separated by 1.772 GHz, corresponding respectively to the D_2b_ and D_2a_ transition groups. The four ^2^P multiplets (F = 0, 1, 2 and 3) are separated by only 16, 34, and 60 MHz, respectively. These levels are themselves divided into a number of different M states and various selection rules determine which (F, M) transitions can occur between the upper and lower levels. Sodium D_2_ line fluorescence spectrum in mesospheric layer is depicted in Fig. 1(b). At mesospheric temperatures near 190 K, the D_2a_ and D_2b_ lines are Doppler broadened to about 1 GHz each full width at half maximum (FWHM), giving rise to the characteristic double-hump absorption profile^28–30^. The D_2a_ transition from F = 2 ground state is usually chosen in LGS application for higher oscillation strength. Once an atom is in the F = 1 state, it can only be excited if D_2b_ line is pumped as well, which is known as repumping. Theoretical simulation for the effectiveness of D_2b_ repumping have been widely studied and show that the return flux can be boosted by the simultaneous excitation of the sodium D_2a_ and D_2b_ lines with 10–20% of the laser power in the latter^31–33^. Besides, if the atom is excited with circularly polarized light, only transition with ΔM is either +1 (right hand) or −1 (left hand) is permitted, whereas with linearly polarized light, only ΔM = 0 is permitted^34^. The atoms cycle on the transition from 3^2^S_1/2_ (F = 2, M = 2) to the 3^2^P_3/2_ (F = 3, M = 3) induced by ∆M = 1 circularly polarized light resonant has the highest transition cross section, and one such transition is indicated by the solid line in Fig. 1(a). The use of circularly polarized laser is proposed to have brighter guide star utilizing the optical pumping process. The increase in return photons for circularly over linearly polarized light has been reported both experimentally and theoretically^35^.

**Figure 2:**
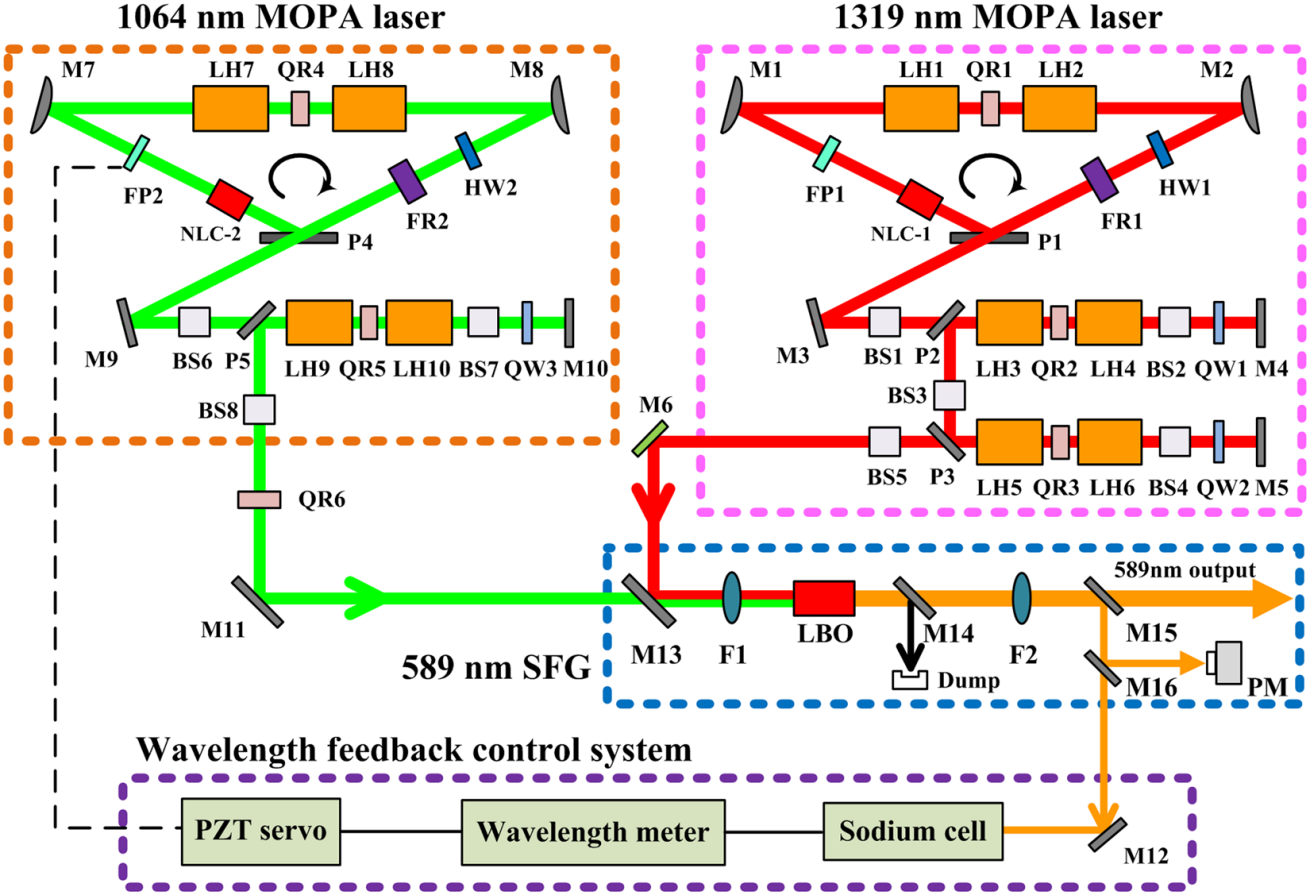
Schematic of the overall experimental layout for sodium laser. LH1-LH10, laser head; QR1-QR6, quartz rotator; M1-M12, high reflector; P1-P5, thin film polarizer; HW1-HW2, half-wave plate; FR1-FR2, Faraday rotator; FP1-FP2, etalon; NLC, nonlinear crystal; BS1-BS8, beam shaper; QW1-QW3, quarter-wave plate; M13, HR at 1319 and AR at 1064 nm; M14, filter; M15 and M16, AR at 589 nm; PM, power monitor/meter; F1-F2, lens.

The experimental configuration for sodium D_2_ radiation in extra-cavity single-pass SFG is illustrated in Fig. 2. There are four subsystems: a 1064 nm MOPA laser^36^, a 1319 nm MOPA laser, a SFG unit, and a wavelength feedback control system. In general, the seed lasers employ three-mirror unidirectional ring cavities with the only difference being optical coatings. It contains two reflectors, a polarizer, a nonlinear crystal, an etalon, two Nd:YAG laser heads, a 90° quartz rotator, a half-wave plate, and a Faraday rotator. The combination of the polarizer, the half-wave plate, and the Faraday rotator makes the ring laser unidirectional operation to eliminate spatial hole. The etalon is introduced to narrow the laser linewidth and tune the central wavelength precisely. The nonlinear crystal is employed as frequency doubler to suppress relaxation oscillations^37^, thus eliminating the saturation effect of sodium atoms absorption to improve the return flux efficiency. Temperatures of the etalons and crystals are accurately maintained within ± 0.05 °C. The 1064 nm oscillator is remotely angle-tuned with a PZT-controlled etalon to allow the SFG laser frequency to be set at the sodium D_2a_ resonance at any time, using feedback from the wavelength control system. The output beam of each oscillator is injected into its respective amplifier after beam shaper. Each amplifier consists of two Nd:YAG gain heads which amplifies the pulse train up to the power required. The 1319 nm amplifier is the same as the 1064 nm amplifier except for coatings and the additional second-stage double-pass amplifier account for the lower gain of Nd:YAG at 1319 nm. The laser heads mentioned above are identical side-pumped by QCW pulsed diode arrays. The 1064 and 1319 nm laser beams after amplification are mode matched into a LBO nonlinear crystal for single-pass 589 nm SFG after collimation. The 4 × 4 × 60 mm^3^ LBO crystal is antireflection coated for all three wavelengths on both facets, and oriented for type-I noncritical phase-matched operation at about 40 °C without walk-off effect. With a high-finesse wavelength meter and a PZT PID controller of the 1064 nm seed laser, the central wavelength of the 589 nm output can be finally locked to sodium D_2a_ absorption line.

**Figure 3:**
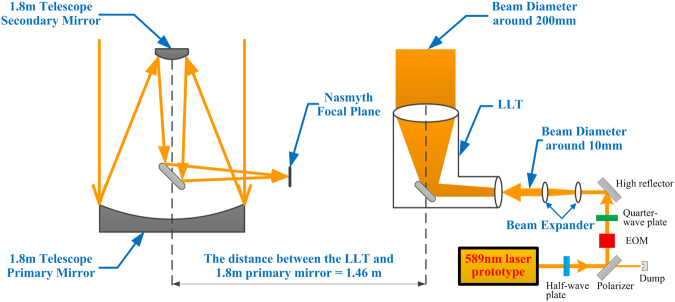
Schematic diagram of the experiment of the laser on-sky test.

The laser sky-test schematic diagram is illustrated in Fig. 3. The 589 nm beam is of a vertical polarization output with beam size of around 3 mm. A power attenuator is composed of a half-wave plate and a polarizer. By controlling the rotation angle of the half-wave plate, one could change the emitted laser power afterward with still maintaining the vertical polarization direction as required by an electro-optic modulator (EOM). The tranmission light from the polarizer is collected by a beam dump to prevent the laser damage. The EOM is placed after the polarizer and used to create the D_2b_ side-band signal. The power ratio of the side frequency to the total laser power can be continuously adjusted by changing the modulation depth. The frequency separation between the main line and sidebands is tunable by changing the modulation frequency. In this way, the main frequency and sidebands of 589 nm laser are guaranteed to resonate with the sodium D_2_ line simultaneously. A quarter-wave plate is placed at the end to convert the linear polarized light to circular polarized light for optical pumping. Following, we use a beam expander (two lenses) to expand the beam size from 3 to 10 mm, and then transfer the beam to the Laser Launch Telescope (LLT). The LLT can further expand the laser beam from 10 mm to 200 mm and project it to the sky. The 1.8-meter telescope is used as the receiving telescope, and a CCD is mounted at the Nasmyth focal plane to measure the magnitude and the photon return of the sodium beacon. The distance between the optical axes of the launch telescope and the receive telescope is nearly 1.46 m, and this distance is enough to separate the Rayleigh scattering and the sodium LGS.

## Results and Discussion

### Characteristics of sodium pulsed laser prototype

**Figure 4:**
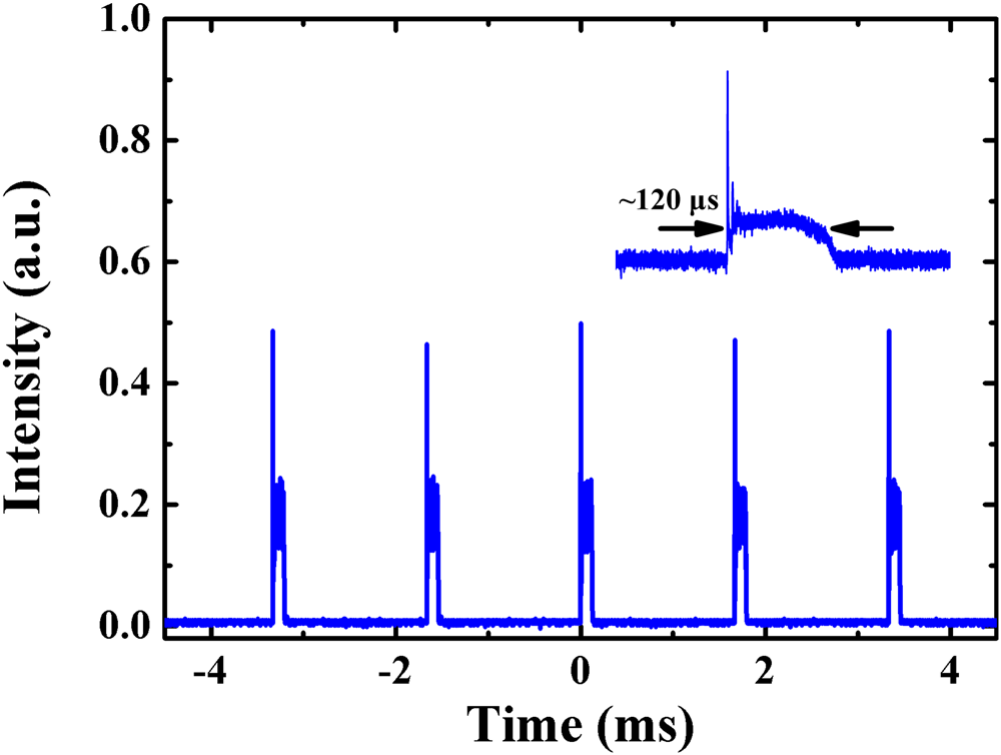
Pulse train of the 589 nm laser. Inset: expanded single pulse profile.

**Figure 5:**
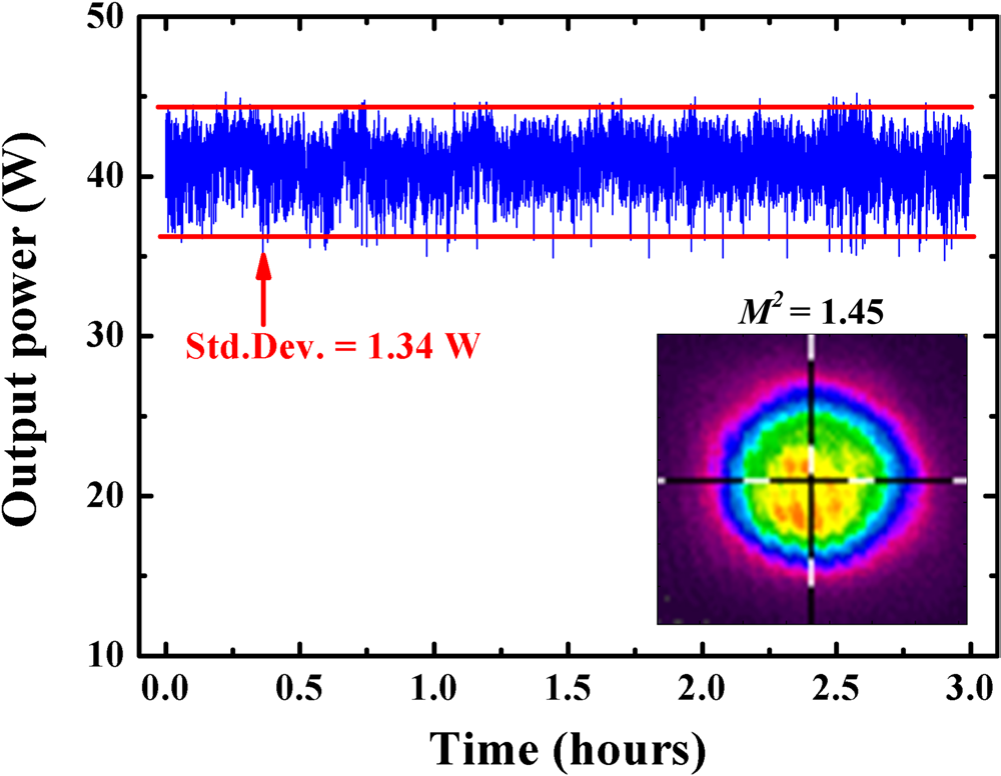
Power-stability test of the sodium laser prototype over 3 h. Inset: 2D beam intensity profile of the laser.

**Figure 6:**
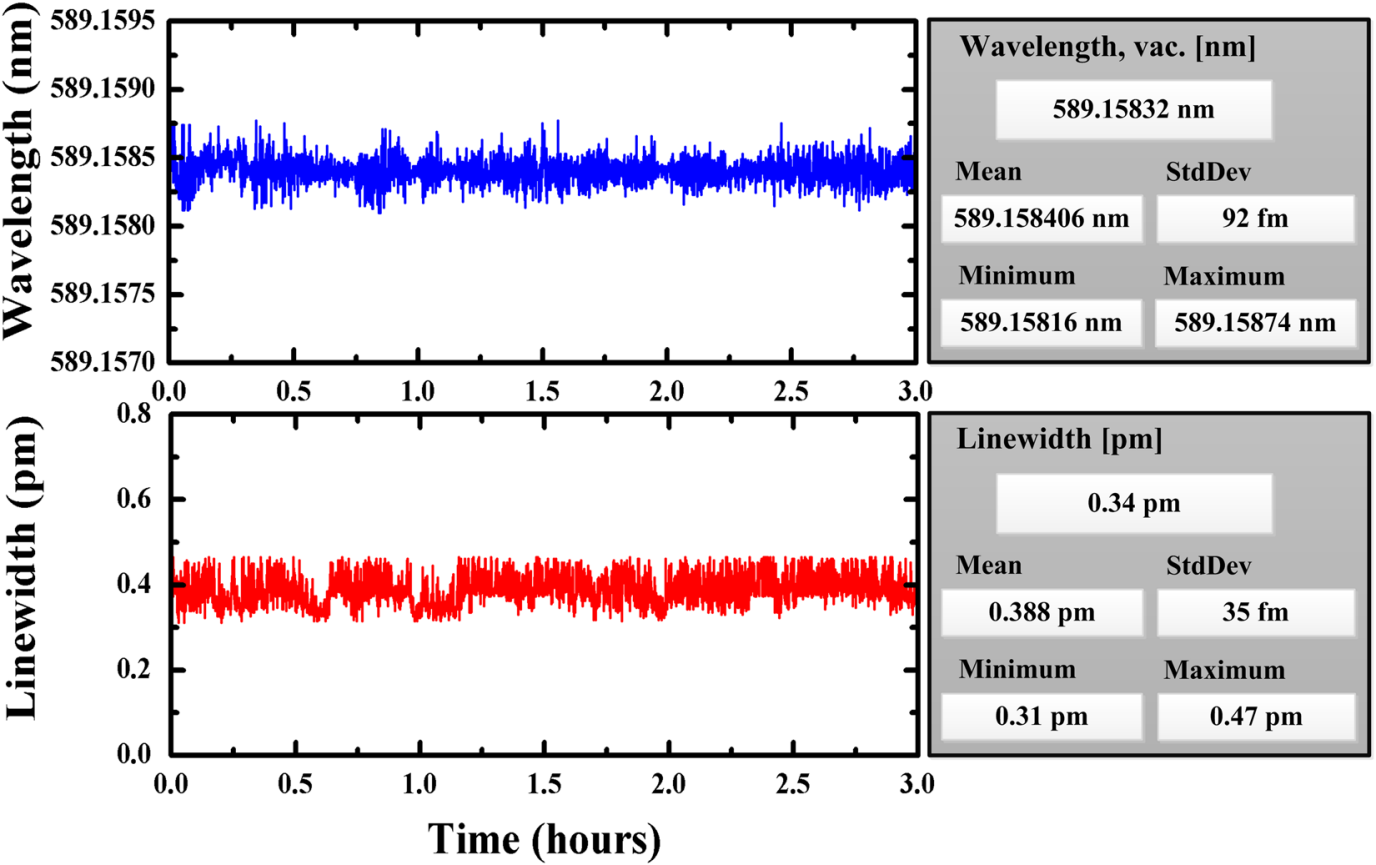
Wavelength-stability and linewidth test of the laser located at 589.1584 nm of sodium D_2a_ line over 3 h.

A summary of the sodium laser prototype properties and some principal design parameters is listed in Table 1. The sodium laser could produce tunable repetition rate from 400 Hz to 1000 Hz with hundred-microsecond level pulse duration, by changing the pulse repetition rate of diode laser power supply for 1064 and 1319 nm beams. These regimes of pulse duration and repetition rate are of interest for the astronomical LGS AO system to better eliminate the problem of the Rayleigh scattering noise from low atmosphere. A typical pulse train of the 589 nm laser is shown in Fig. 4, and it depicts the pulse repetition frequency is 600 Hz with a pulse duration of 120 µs. It is noticeable from the inset that, except for the initial overshoot, the 589 nm pulse shape with a doubler is smooth without relaxation oscillation spikes, in comparison with high-amplitude spikes without a doubler in ref.^19^, which will enhance the LGS return efficiency due to avoid the saturation effect of sodium atoms absorption induced by the high spiking peak power. The power output of the 1064 nm and 1319 nm lasers, measured shortly after the integration of the laser, are found to be 90.2 W and 67.5 W, respectively. The yellow 589 nm output power is measured to be as high as 41 W, which implies an optical power conversion efficiency in the SFG stage of 26%. For the LGS observations, the long-term power and wavelength stability of sodium laser are the key performance parameter. Because the laser power cannot be measured simultaneously with the data acquisition of the CCD camera, a beam splitter inserted in the prototype light path diverts approximately 0.1% of the 589 nm laser light to the power monitor, and the laser power value given in this paper is the converted measurement of inside monitor power. As shown in Fig. 5, the power stability at the output power of 41 W is monitored to be within ± 3.3% over 3 h, using a power meter (OPHIR, FL400A-LP1–50). The linear s-polarization ratio of the output is better than 100:1. The inset of Fig. 5 shows the two-dimensional (2D) beam spatial profile of the laser with a beam quality of *M*^2^ = 1.45, indicating that the 589 nm laser operates in perfect Gaussian mode. With a servo for high speed negative feedback loop to control the wavelength of the 1064 nm seed, the wavelength stability and linewidth of the sodium laser are monitored with a wavelength meter (WS-7). As shown in Fig. 6, the wavelength deviation is determined to be better than ± 0.29 pm for 3 h and the average value of linewidth is 0.388 pm, corresponding to a frequency fluctuation of ± 0.25 GHz with a line width of less than 0.335 GHz.

**Table 1:** Main parameters of the 41 W sum-frequency laser prototype.

Attribute	Value or Range
Average Power at 589 nm	41 W@600 Hz
Average Power at 1064 nm	90.2 W@600 Hz
Average Power at 1319 nm	67.5 W@600 Hz
Sum-frequency efficiency	26%
Line Width	~0.34 GHz
Beam Quality	*M*^2^ ~ 1.45
Pulsed Length	100–180 μs
Repetition Rate	400–1000 Hz
Beam line polarization ratio	>99%

The sodium laser prototype is transported to the Lijiang Observatory of China at an altitude of 3300 m, and installed on the 1.8 m telescope to test the LGS in collaboration with the Institute of Optics and Electronic, Chinese Academy of Sciences. The coordinate of the test site is 26.7042°N 100.0370°E. The zenith and azimuth angles of the telescope pointing are 21.2° and 10.2°, respectively, and the location of the parallel direction of the geomagnetic lines is 41.4°. On the nights of 2017 May, a test campaign is conducted. The transform loss from the laser machine to the outcome of the LLT is estimated to be approximately 17%.

### Eliminating Rayleigh scattering

**Figure 7:**
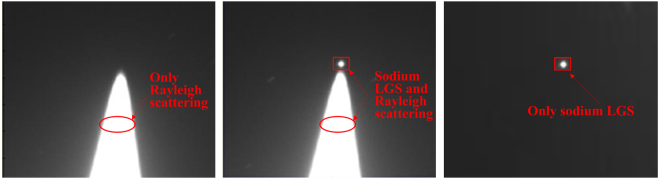
Images of the sodium LGS and Rayleigh scattering. (**a**) only Rayleigh scattering when the wavelength of laser prototype is walked away from sodium resonance frequency; (**b**) sodium LGS occurs when the wavelength of laser prototype is tuned to the sodium resonance frequency; (**c**) only sodium LGS after eliminating the Rayleigh scattering.

Initially, the wavelength of yellow laser output is walked away from sodium resonance frequency. Figure 7(a) shows that there is only Rayleigh scattering catched by the Nasmyth CCD. Then, sodium LGS occurs when the wavelength of yellow laser is tuned to the sodium resonance frequency, as shown in Fig. 7(b). Because the sodium LGS spot is close to the Rayleigh scattering, it is necessary to eliminate the strong Rayleigh scattering noise. Unlike all other CW operating sodium LGS AO systems, the pulse format of the laser provides the ability to gate Rayleigh and other scattered light from the wavefront sensor. Rayleigh scatter comes from lower altitudes and thus returns more quickly than the light from the sodium layer. With this pulse format, all the Rayleigh scatter created by a pulse returns to the ground before the first sodium return. Thus, the Rayleigh scattering can be cut off by controlling the shutter on/off of CCD synchronized to the received fluorescence time from the layer of atomic sodium in the mesosphere at 80–100 km altitude. As a result, only sodium LGS is displayed in Fig. 7(c). The LGS spot size on sky is minimized by adjusting the focal stage distance of secondary mirror of LLT. A 4″ (FWHM) angular size LGS spot with an exposure time of 100 ms is achieved.

### Optimization of yellow wavelength

**Figure 8:**
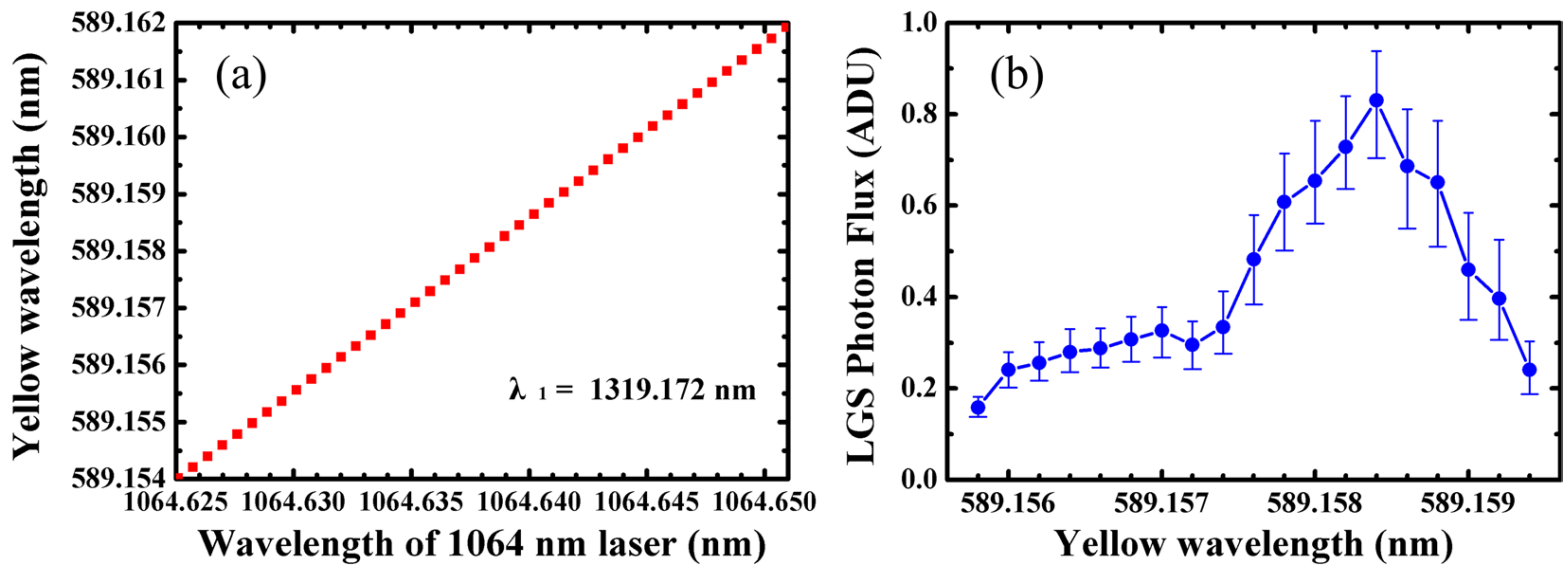
(**a**) Wavelength tuning around sodium resonance frequency and (**b**) photon flux of sodium atom on sky.

After getting the sodium LGS image, we optimize the sodium laser prototype to find the location of the wavelength for the highest photo return. Because the temperature of the sodium mesospheric is quite lower than the laser lab ambience temperature, the best middle frequency will be a little different between the mesospheric and the sodium vapor in the cleaning room. In our case, the precise wavelength tuning of the sodium laser is accomplished by using the solid etalons in each oscillator. One wavelength is fixed at 1319.172 nm. While the temperature of the etalon in 1064 nm oscillator changes from 40 °C to 43.6 °C with an increment of 0.1 °C, the 1.06 μm wavelength is linearly tuned from 1064.625 to 1064.651 nm in a step-length of about 0.73 pm. As plotted in Fig. 8(a), this tuning region corresponds to the tuning region of the yellow output wavelength from 589.15398 to 589.16211 nm in 0.25 pm steps, which cross the 3-GHz Doppler-broadened absorption width of mesospheric sodium. Moreover, it is observed that there are no significant changes in output power around 589 nm as the wavelength tuning. Meanwhile, a series of images at different yellow center wavelength are taken by CCD. Figure 8(b) exhibits the average photon flux of sodium LGS as a function of the wavelength. The maximum photon flux is located at 589.1584 nm for the sodium D_2a_ line. Returned photon gradually decreases with detuning from 589.1584 nm. The other maximal value of photon flux could be found as a shoulder of above curve near the wavelength of 589.1568 nm for the sodium D_2b_ line. To keep the laser working at a stable state, the central wavelength of the laser prototype output is locked to the sodium D_2a_ absorption line at 589.1584 nm during the all on-sky test for about three hours.

### Comparison for linear and circular polarization

**Figure 9:**
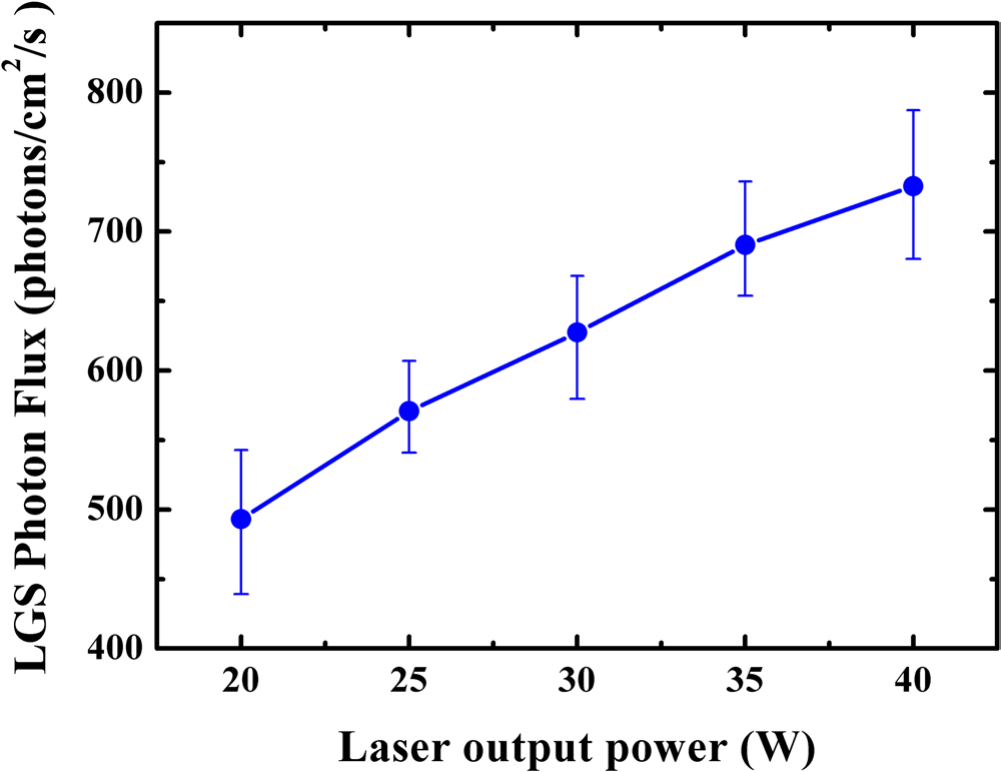
Measurement of photon return as a function of sodium laser output power for linearly polarized beam.

**Figure 10:**
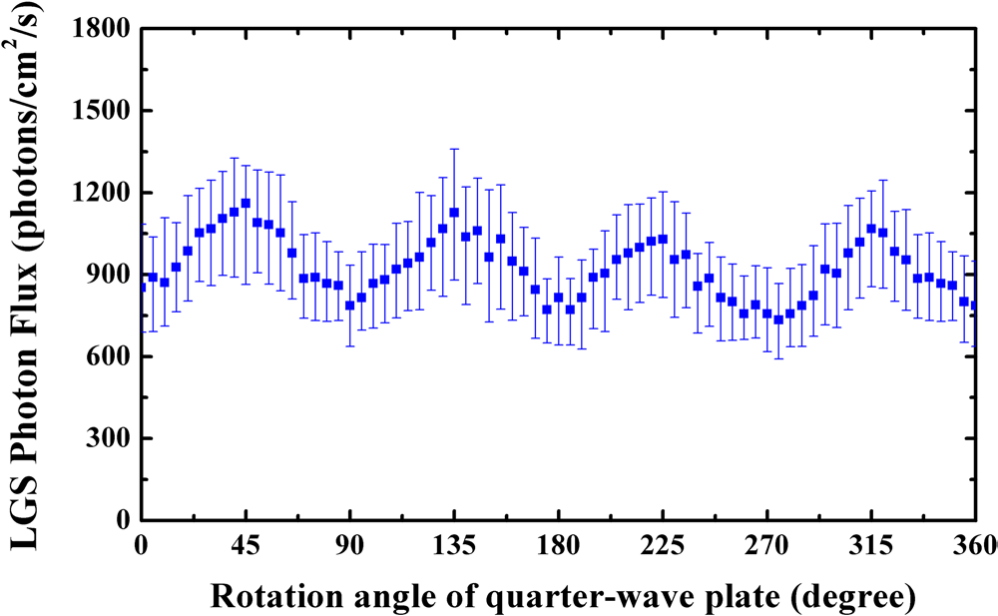
Measurement of photon flux of the sodium beacon as a function of rotation angle of the quarter-wave plate. The circular polarized light comes out when the angle of the plate is 45°, 135°, 225°, and 315°, respectively.

Since the wavefront detection performance of AO is directly related to the brightness of LGS, it is important to maximize its photon generation efficiency by all means. Figure 9 shows the LGS returned flux under different laser output power for a linearly polarized beam. It can be seen that the LGS flux grows monotonically with the increasing prototype power from 20 to 41 W, and does not show any saturation effect up to the maximum pump power, which indicates that higher flux can be achieved with higher launch energy. As mentioned above, circular polarized beams have the capability to increase sodium photon return. Figure 10 shows the return photons of sodium LGS at different rotation angle of quarter-wave plate. The observed variation is fit with a sinusoid in order to determine the optimal rotation angle. When the angle of the plate is set to 0, 90, 180, 270, and 360°, where the fast axe of plate is oriented at parallel or vertical to the yellow beam polarization direction, and a minimal photon flux of about 770 photons∕cm^2^∕s is generated for linearly polarized light. Circular polarization is obtained by rotating the angle of the plate to be 45, 135, 225, and 315°, respectively, where the maximal value of photon flux of about 1090 photons∕cm^2^∕s is achieved. It can be seen that circular polarized light will bring approximate 42% or even more returned photons than linearly polarized light during the tests.

### Effectiveness of D_2b_ repumping

**Figure 11:**
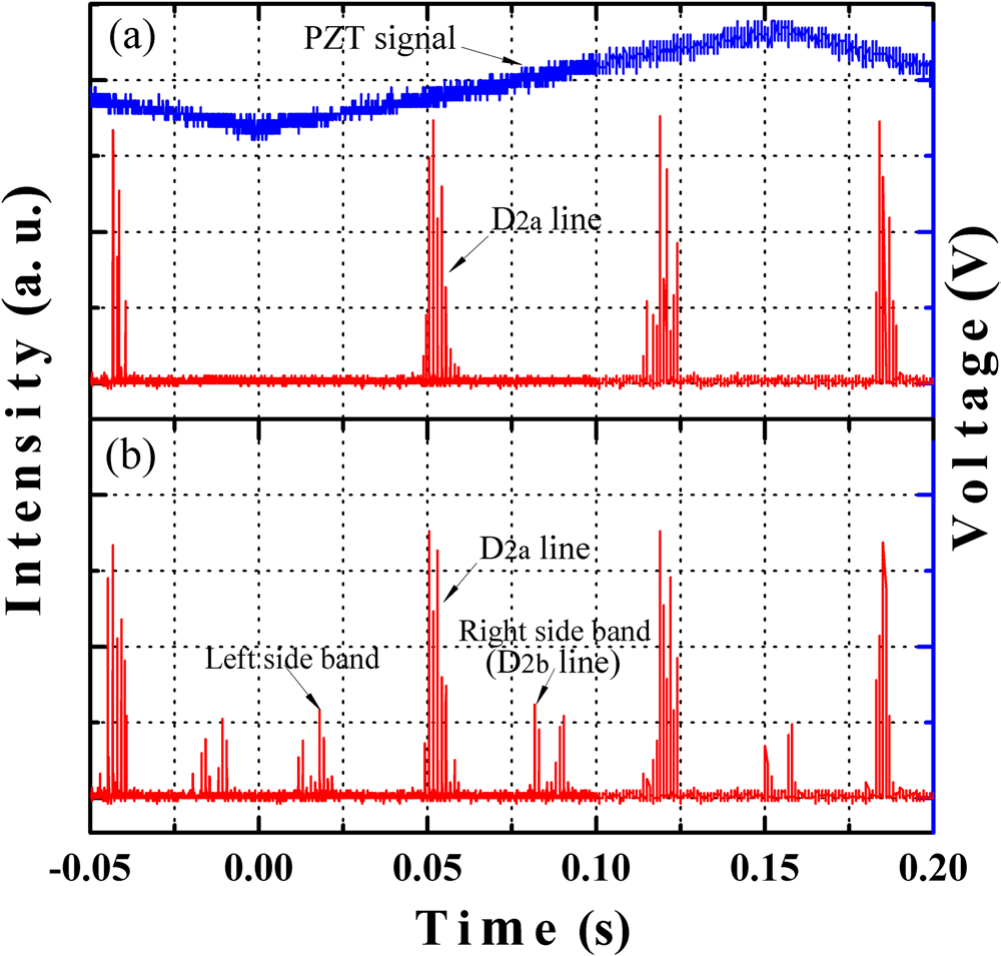
Spectrum of the 589 nm laser measured with a Fabry-Perot interferometer. (**a**) with the EOM off: D_2a_ line; (**b**) with the EOM on by allocating 10% of the laser power to the D_2b_ line: two symmetrical sidebands around D_2a_ line.

Moreover, repumping of 10–20% of the total laser power on the D_2b_ line is suggested as an effective method of enhancing the photon return efficiency. In the experimental study, the sodium D_2b_ repumping is implemented by applying a 1713 MHz resonance frequency signal to the EOM. The spectra of the 589 nm output are measured by means of a Fabry-Perot interferometer with 3.75 GHz free spectral range and 25 MHz frequency resolution. As shown in Fig. 11(a), only sodium D_2a_ line is observed in the laser spectrum when the EOM is off. With the EOM on, there are two symmetrical frequency components (left sideband and right sideband) that are 1.71 GHz away from the 589 nm main laser line (D_2a_ line), as shown in Fig. 11(b). The blueshifted sideband (shift  +1713 MHz corresponding to 589.1568 nm) is going to repump the sodium atoms at the D_2b_ line for the LGS system and its amplitude can be continuously adjusted with respect to the main line. For the LGS application, the required power ratio between the D_2b_ to D_2a_ lines is around 10%. At the same time, another 10% of the total power is converted to the redshifted sideband (shift −1713 MHz), which will not interact with the sodium atoms. Thus the laser power in the D_2a_ line is reduced by 20% when the EOM is on.

**Figure 12:**
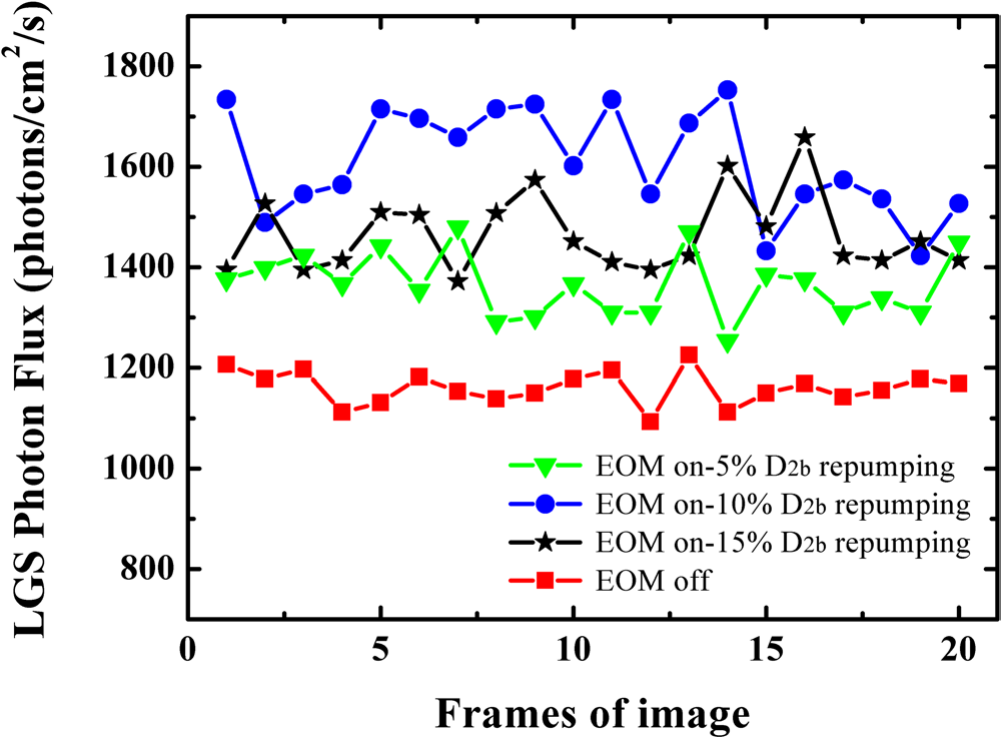
Measurement of photon return without and with 5%, 10% and 15% D_2b_ repumping for 41 W output power of the sodium LGS laser.

By changing the modulation intensity of the EOM, the 5%, 10% and 15% of yellow power shifted to D_2b_ is adjusted and investigated, respectively. Figure 12 gives the LGS returned flux when the EOM is turned on and off at 41 W output power. For each set of measurements, 20 frames of images are taken. In most cases, standard deviation of the calibrated flux of sodium beacon is less than 7%. This variation contains the drift of output laser power, changes of the atmospheric transmission, sodium layer activity, and the calibration error of sodium beacon. As seen from Fig. 12, a significant improvement in the photon return is demonstrated with EOM for D_2b_ repumping. An average maximum of 39% photon return enhancement than that without repumping is observed when 10% of yellow power is detuned to D_2b_ repumping for a circular polarized beam, which agrees well with the numerical simulation result^21^. As a final result for this field test, a maximum average return flux of sodium LGS is measured to be 1610 photons/cm^2^/s while using a circular polarized light and output power of the laser is 41 W with 10% D_2b_ repumping, corresponding to ~47 photons/cm^2^/s/W of emitted laser power. This demonstration represents a critical step toward addressing the need of a high brightness sodium LGS for a QCW μs-pulse sodium laser. Our next plan is to use a sodium lidar system in parallel with the testing of sodium LGS, to monitor the sodium column density, a parameter needed for determination of the laser coupling efficiency.

## Conclusion

In conclusion, a QCW μs-pulse 589 nm sodium laser with tunable repetition rate (400 Hz to 1 kHz) is demonstrated based on sum-frequency mixing two amplified Nd:YAG ring lasers in LBO. Here, we present results of on-sky test performed at Lijiang Observatory to characterize the performance of this prototype laser for AO. The laser operates at a pulse repetition rate of 600 Hz with an output power of 41 W. The suppression of laser spiking is realized by intracavity second harmonic generation. The central wavelength of the laser output is accurately locked to the sodium D_2a_ absorption line. The increase of the photon return is around 42% when use the circular polarization to instead of the linear polarization. Moreover, the laser also emits an adjustable sideband at 1.71 GHz away from the main laser frequency for better sodium-D_2b_ excitation, where the sodium laser with 10% of total power on D_2b_ repumping will bring 39% photon return increases than that without D_2b_ repumping. In the best test case, a sodium beacon at a spot size of 4″ is successfully observed with maximum return flux up to 1610 photons∕cm^2^∕s (i.e. ~47 photons∕cm^2^∕s/W).
